# Comparing the Nucleocapsid Proteins of Human Coronaviruses: Structure, Immunoregulation, Vaccine, and Targeted Drug

**DOI:** 10.3389/fmolb.2022.761173

**Published:** 2022-04-29

**Authors:** Bo Zhang, Junjie Tian, Qintao Zhang, Yan Xie, Kejia Wang, Shuyi Qiu, Keyu Lu, Yang Liu

**Affiliations:** ^1^ College of Basic Medicine, Zunyi Medical University, Zunyi, China; ^2^ School of Public Health, Zunyi Medical University, Zunyi, China; ^3^ Key Laboratory of Plant Resource Conservation and Germplasm Innovation in Mountainous Region (Ministry of Education), College of Life Sciences/Institute of Agro-bioengineering, Guizhou University, Guiyang, China

**Keywords:** coronaviruses, nucleocapsid protein, structure, immunoregulation, vaccine, targeted drug

## Abstract

The seven pathogenic human coronaviruses (HCoVs) include HCoV-229E, HCoV-OC43, HCoV-NL63, and HCoV-HKU1, which usually cause mild upper respiratory tract diseases, and SARS-CoV, MERS-CoV, and SARS-CoV-2, which cause a severe acute respiratory syndrome. The nucleocapsid (N) protein, as the dominant structural protein from coronaviruses that bind to the genomic RNA, participates in various vital activities after virus invasion and will probably become a promising target of antiviral drug design. Therefore, a comprehensive literature review of human coronavirus’ pathogenic mechanism and therapeutic strategies is necessary for the control of the pandemic. Here, we give a systematic summary of the structures, immunoregulation, and potential vaccines and targeted drugs of the HCoVs N protein. First, we provide a general introduction to the fundamental structures and molecular function of N protein. Next, we outline the N protein mediated immune regulation and pathogenesis mechanism. Finally, we comprehensively summarize the development of potential N protein-targeted drugs and candidate vaccines to treat coronavirus disease 2019 (COVID-19). We believe this review provides insight into the virulence and transmission of SARS-CoV-2 as well as support for further study on epidemic control of COVID-19.

## 1 Introduction

Coronaviruses (CoVs) are positive-stranded RNA viruses that taxonomically belong to the family Coronaviridae of Nidovirales (subfamily Coronavirinae), which is subdivided into four genera: α, β, γ, and δ. α-CoV and β-CoV can infect mammals, whereas γ-CoV and δ-CoV generally infect birds. Pathogenic human coronaviruses (HCoVs) include seven members. HCoV-229E, HCoV-OC43, HCoV-NL63, and HCoV-HKU1 usually cause mild to moderate upper airway respiratory cold in humans, whereas SARS-CoV, MERS-CoV, and SARS-CoV-2 can cause severe respiratory diseases (Table 1) (Chan et al., 2020). The number of SARS-CoV-2 replicated copies on the surface of the lung epithelial layer is 100 times that of HCoV-OC43 and 1,000 times that of SARS-CoV (Essaidi-Laziosi et al., 2018). Thus, although the mortality of SARS-CoV-2 is lower than that of SARS-CoV and MERS-CoV, its spreading efficiency worldwide is much higher (Wang et al., 2020).

**TABLE 1:** Seven human pathogenic Coronaviruses.

Virus	Genus	Symptoms	Fatality Rate	Media
SARS-CoV-2	β- Coronaviruses	Severe acute respiratory syndrome	3.70%	human-human,human-animal
SARS-CoV	β- Coronaviruses	Severe acute respiratory syndrome	10%	human-human,human-animal
MERS-CoV	β- Coronaviruses	Severe acute respiratory syndrome	37%	human-human,human-animal
HCoV-HKU1	β- Coronaviruses	Pneumonia	rarely	human-human
HCoV-OC43	β- Coronaviruses	Mild respiratory tract infection	rarely	human-human
HCoV-NL63	α-Coronaviruses	Mild respiratory tract infection	rarely	human-human
HCoV-229E	α-Coronaviruses	Mild respiratory tract infection	rarely	human-human

**FIGURE 1:**
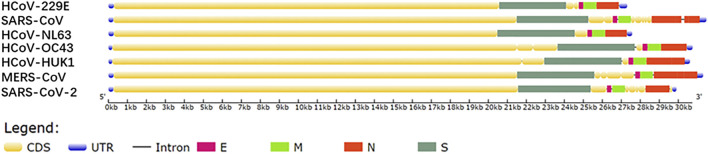
Gene sequences map drawn based on the NCBI database.

All seven HCoVs sequences contain non-coding regions at both ends and complete open reading frame (ORF) that encodes 4 structural proteins, 15 nonstructural proteins (nsp), and 7 kinds of accessory proteins (Chan et al., 2020). Four structural proteins are composed of nucleocapsid protein (N), spike protein (S), membrane protein (M), and envelope protein (E). The genes alignment shows that the ORF gene sequences of the HCoV species are very similar, i.e., replicase, protease ORF1a/b, S, E, M, N (Figure 1) (Chang et al., 2014). Comparing SARS-CoV-2 with six other HCoVs for the four structural protein sequences shows that SARS-CoV-2 and SARS-CoV have the highest sequence identity, between 70 and 95%, and the other five types are around 50% (Table 2).

**TABLE 2:** Gene identity comparison of the seven pathogenic HCoVs.

Virus	GC Content (%)	Sequence Length (kb)	Complete (%)	1ab (%)	E (%)	M (%)	N (%)	S (%)
SARS-CoV-2	38	29.882	100	100	100	100	100	100
SARS-CoV	40.8	29.751	79.09	79.63	93.51	85.2	87.97	72.76
MERS-CoV	41.2	30.111	51.4	53.73	42.97	50.52	50.96	45.48
HCoV-OC43	38.3	27.317	46.04	49.38	42.62	44.49	38.32	38.84
HCoV-HKU1	32.1	29.926	49.93	53.35	42.97	48.52	42.71	43.74
HCoV-229E	34.5	27.553	45.71	50.14	44.14	43.69	39.36	42.25
HCoV-NL63	36.8	30.741	48.51	52.53	40.63	45.39	43.13	43.86

To search potential antiviral drug targets for the treatment of coronavirus disease 2019 (COVID-19), a method was developed to identify four high-case fatality rates potential genomic determinants from CoVs strains by combining advanced machine learning with traditional genomic comparison techniques. Three of four potential regions are located in the N protein (Gussow et al., 2020), which thus emerged as a potentially key protein against the SARS-CoV-2 pandemic.

**FIGURE 2:**
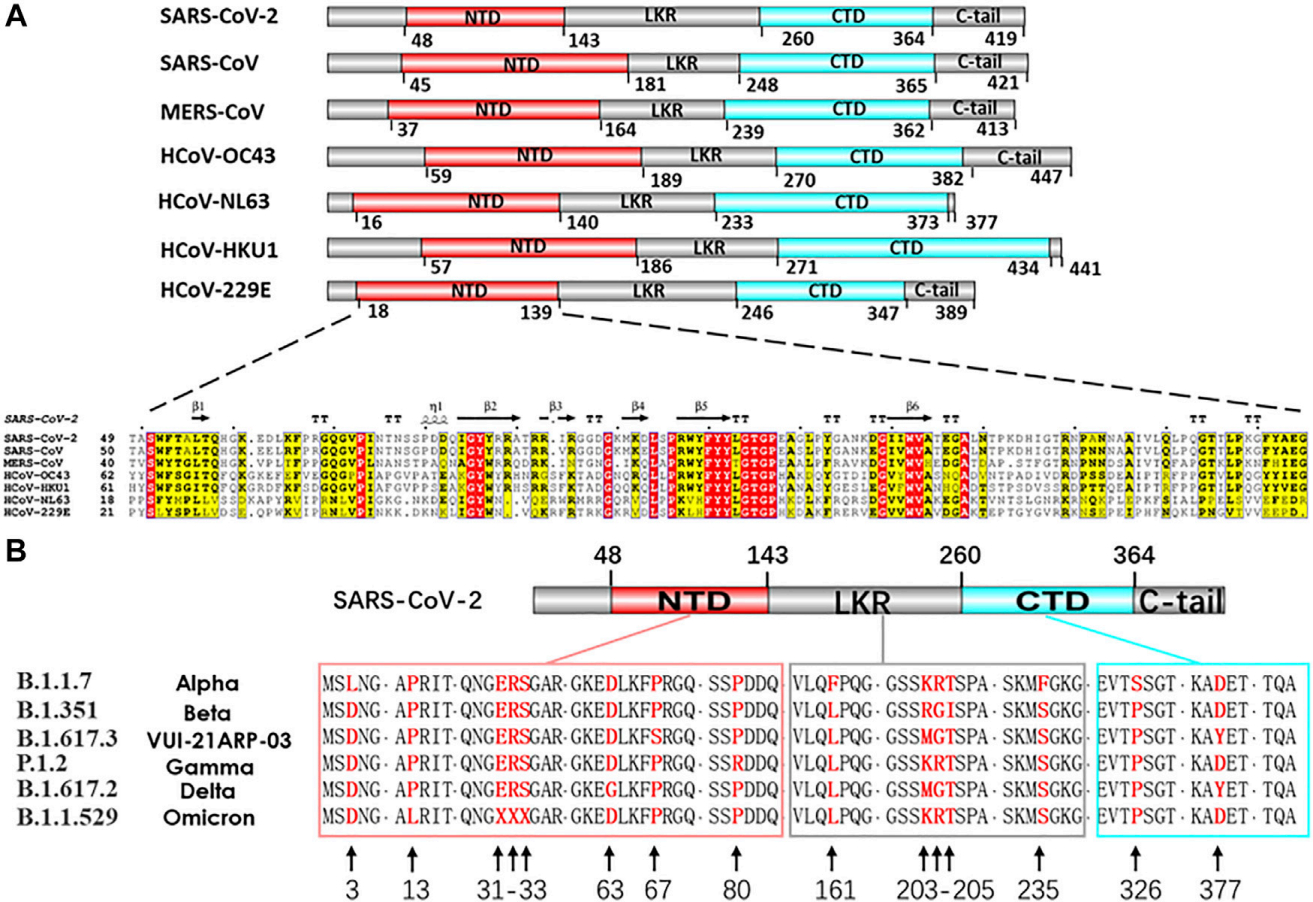
**(A)** Comparison of seven HCoVs N protein structures and sequences alignment of NTD. Nucleocapsid sequences: SARS-CoV-2 (UniProtKB: P0DTC9), SARS-CoV (UniProtKB: P59595), MERS-CoV (UniProtKB: K9N4V7), HCoV-OC43 (UniProtKB: P33469), HCoV-NL63 (UniProtKB: Q6Q1R8), HCoV-HKU1 (UniProtKB: Q5MQC6), HCoV-229E (UniProtKB: P15130). **(B)** In silico analysis of different variants of SARS-CoV-2 N proteins. Nucleocapsid sequences of variants: **(B)**1.1.7 (GenBank: QYU75264.1), **(B)**1.351 (GenBank: QRG27681.1), **(B)**1.617.3 (GenBank: QYV27950.1), P.1.2 (GenBank: QXF22464.1), **(B)**1.617.2 (GenBank: UEA15887.1), **(B)**1.1529 (GenBank: UGY75362.1).

At present, the efforts for developing vaccines and drugs to prevent COVID-19 are primarily focused on the S and N proteins. The structural S protein is the key in determining the specificity of the host cells, the virus pathogenicity, and intermediate transmission. However, the high mutation rate of the S protein gene may weaken the vaccine. Furthermore, the complex molecular process underlying the virus entry into the host cells is mediated by the S protein, which may complicate the vaccine response, as seen before in the HIV 1 Env protein vaccine work (Kwong et al., 2002; Roark et al., 2021). S protein variant strain can escape the targeted therapeutic with different host-cell receptor binding partners (Baral et al., 2021; Garcia-Beltran et al., 2021; Hoffmann et al., 2021; Roessler et al., 2021). The limitations of S protein targeting only antiviral approaches become increasingly prominent. The N protein is another critical structural protein for coronaviral lifecycle, Which acts as the crucial modulator in viral genomic packing, replication and cell signaling pathways (Malone et al., 2022). It is also one of the most conservative functional proteins among CoV evolution (McBride et al., 2014). The N protein gene is more conserved and stable than the S protein, with 90% amino acid homology and fewer mutations over time (Grifoni et al., 2020) (Figures 2A,B). The genetic stability and conservation of the N protein emphasize the advantages of N protein based resistance and durability in candidate drugs and vaccine screening.

This review summarizes the fundamental research studies related to basic structure, immune regulation and therapy on seven HCoV N proteins in recent years and provides ideas for fighting the COVID-19 pandemic. We first focus on the structural composition and universal functional characteristics of the seven HCoVs, and propose a self-assembly model for the formation of ribonucleoprotein (RNP). We then elucidate the HCoV N proteins regulation in immune mechanisms and cellular signaling pathway. We conclude with a discussion of the potential superiority of N protein present in vaccines, the suitability of universal CoV candidate drugs for COVID-19 therapy and the advancement of novel silico screening of SARS-CoV-2 antivirus drugs.

## 2 Structure and Functions of Human Coronaviruses N Proteins

The main function of the N protein is to package the viral genome into a RNP particle to protect the genomic RNA and incorporate it into a viable virion. A single RNP has a reverse G-shaped structure with a diameter of about 14 nm and a height of about 16 nm, and 30–35 RNPs are closely arranged to form a virus particle. *In situ* cryo-electron tomography analysis revealed that the average diameter of SARS-CoV-2 virus particles is 89.8 nm, and each virus particle contains 4.4 × 10^3^ N proteins on average (Macneughton and Davies, 1978; Klein et al., 2020).

**FIGURE 3:**
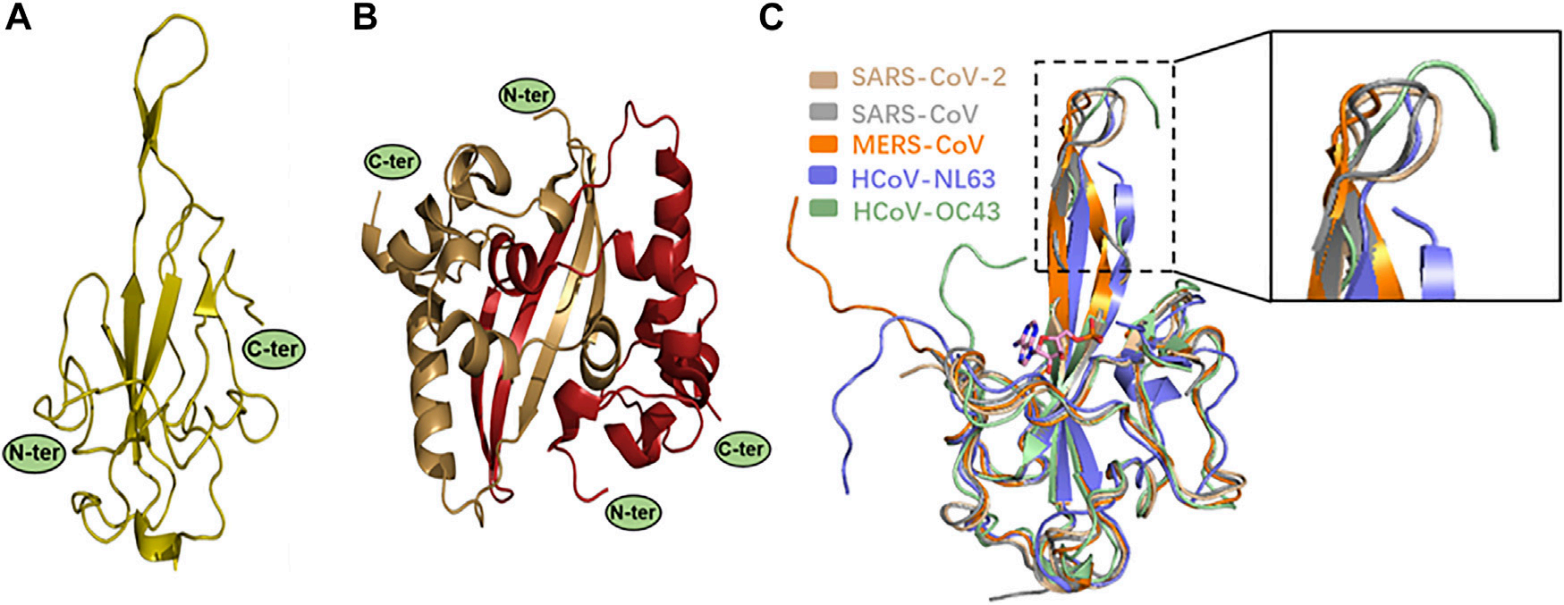
**(A–B)** X-ray structures of SARS-CoV-2 N protein. Monomeric NTD (PDB: 7CDZ) and dimeric CTD (PDB: 7CZ0) are presented in left and right panels, respectively. **(C)** Structural comparison of SARS-CoV-2 N-NTD with other four HCoV N-NTDs. Each structure is denoted by different colors (SARS-CoV, gray, PDB: 2OFZ; SARS-CoV-2, wheat, PDB: 2OFZ; HCoV-OC43, pale green, PDB: 4LI4; MERS-CoV, orange, PDB: 4UD1; HCoV-NL63, slate, PDB: 5N4K). The circles indicate the significant differences, as shown in a zoomed-in panel.

The virus RNA genome itself needs to be easily dissociated and exposed for efficient transcription and replication in the process of infection. The regulatory role of N protein in this process is gradually unraveled as the structural research of N protein makes progress (Masters, 2006; Surjit and Lal, 2008). CoVs N proteins contain three distinct and highly conserved domains based on amino acid sequence comparisons: the N-terminal domain (NTD), C-terminal domain (CTD), the inherent disordered central linker region (LKR), and non-conserved disordered C-terminal region (C-tail) (Cubuk et al., 2021) (Figure 2A, Figure 3A). It should be noted that all of the three domains have been demonstrated to bind with viral RNA, but NTD is the main domain responsible for this task, and CTD is mainly responsible for the dimerization (Surjit and Lal, 2008). The LKR includes a Ser/Arg-rich region (SR-motif) that contains phosphorylation sites (Peng et al., 2008); LKR is responsible for regulating the binding activity of NTD and CTD to RNA, CTD oligomerization, and other processes.

### 2.1 NTD’s High Affinity for RNA

Kang et al. were the first to report the NTD crystal structure of SARS-CoV-2 N protein (N-NTD). Its overall structure is similar to the other reported CoV N-NTDs. However, they found a unique potential RNA binding pocket, and its surface electrostatic potential characteristics are also unique (Figure 6B). The significant changes on the surface characterizations of this region, which may result in the RNA-binding cleft being adaptive to its genome RNA, help to identify its genome RNA (Kang et al., 2020). Peng et al. also analyzed the SARS-CoV-2 N-NTD crystal structure and compared it with other HCoV N-NTDs. They found that the N-NTDs from the highly contagious viruses, namely, SARS-CoV-2 (wheat), SARS-CoV (gray), and MERS-CoV (orange), have an N-terminal loop extending outward as a protruding and flexible β-hairpin domain, while in the weakly contagious viruses, namely, HCoV-OC43 (pale green) and HCoV-NL63 (slate), the N-terminal loop of the N-NTD rotates toward the core subdomain, and no flexible connecting loop is seen in the structure (Peng et al., 2020a). This flexible loop improves the N protein and RNA binding and may be one of the reasons for the great difference in pathogenicity between different CoVs (Figure 3C). Studies have reported a molecular structure model of the binding of HCoV-OC43 N-NTD to adenosine monophosphate (AMP) in which residues S64, Y126, G68, and R164 bind to AMP through hydrogen bond interactions, and residue Y124 further stabilizes the binding through π-π stacking (Lin et al., 2014). By comparing the residues involved in AMP binding between SARS-CoV-2 and HCoV-OC43 N-NTDs, these five residues also proved to be related to RNA binding in SARS-CoV-2 (Kang et al., 2020). In addition, the RNA level of the matrix protein had a seven-fold increase in cells transfected with plasmids encoding the mutant N protein (R106A) compared to cells transfected with plasmids encoding the wild-type protein. It was proposed that these six residues may be the target binding sites of N protein and RNA (Chen et al., 2013). By comparing these six residues’ positions in the seven HCoVs, it was found that they are highly conserved in the β-CoVs, and relatively not conserved in the α-CoVs (HCoV-NL63 and HCoV-229E); this may also be another reason for the greater difference in infectivity between α-CoVs and β-CoVs. Subsequently, by studying the crystal structure of HCoV-NL63 N-NTD, it was found that the binding site was indeed distinct from that of HCoV-OC43 N-NTD (Szelazek et al., 2017), which further proved the difference between the N-NTDs. However, the nucleic acid-binding domain of HCoV-NL63 is mainly located at residues 2–144 and is similar in α-CoVs and β-CoVs (Zuwała et al., 2015). To explore specific amino acid binding positions, it may be necessary to analyze further the three-dimensional structure of the composite crystal of SARS-CoV-2 N protein and RNA.

### 2.2 C-Terminal Domain Is Responsible for the Oligomerization

Crystal structure analysis of the SARS-CoV-2 N-CTD showed that the N protein exists as a tight homodimer with a rectangular slab (Figure 3B) (Peng et al., 2020a). In other HCoVs, the full-length N protein also exists as a dimer, and its formation process is closely related to the N-CTD domain (Jayaram et al., 2006; Chen et al., 2007; Huang et al., 2009; Lo et al., 2013). It is worth noting that the SARS-CoV-2 N protein exists as a high-order polymer in normal saline-containing RNA, similar to the SARS-CoV, HCoV-229E, and HCoV-OC43 (Chang et al., 2013), indicating that the dimer is probably only an intermediate state in the process of virus replication. A series of truncated versions of the SARS-CoV-2 N protein were purified and residues (247–419) were found to form homotetramers in solution, while individual residues (247–364) form dimers, and the C-terminal disordered domain residues (365–419) only exist as monomers and dimers (Ye et al., 2020). Therefore, it is proposed that the self-assembly process of the SARS-CoV-2 N protein is first mainly based on dimerization by the N-CTD domain (247–364). In the process of RNA binding to SARS-CoV-2 N protein, the protein structure changes to expose the disordered C-terminal region on the molecule surface. The disordered C-terminal region residues (365–419) mediated homotetramerization and further formed higher-order polymers.

### 2.3 Linker Region Regulates Multiple Processes

LKR is a multi-functional region, including an important phosphorylation site (SR). If the gene encoding SR is deleted in the SARS-CoV N protein, it will lead to a reduction of the transcription levels and the numbers of infectious virus particles significantly are also reduced (Tylor et al., 2009). The deletion of residues 184–196 eliminated the self-polymerization of N protein, indicating that the SR motif is very important for this process (He et al., 2004). The LKR region has also been found to interact with nucleic acid and M protein directly (Takeda et al., 2008; Chang et al., 2009). Yeast two-hybrid and surface plasmon resonance analysis have also revealed that the SARS-CoV N protein’s C-tail residues (365–419) interact with M protein residues (197–221) through electrostatic attraction (Luo et al., 2006). The participation of M protein may further promote the self-assembly of spiral RNP.

**FIGURE 4:**
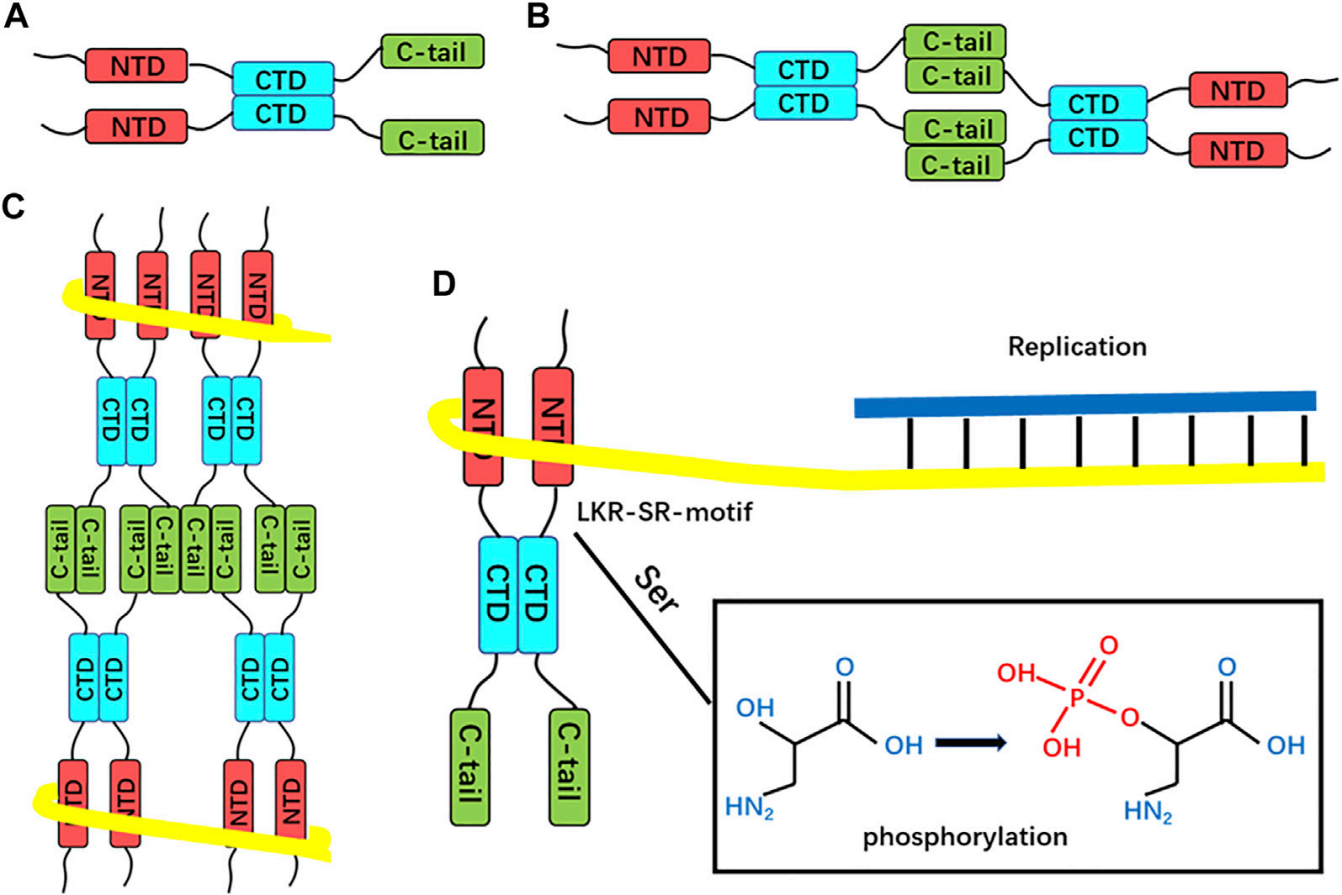
Proposed SARS-CoV-2 N protein-mediated RNP packaging mode. **(A)** Dimerization is mediated by the CTD domain. **(B)** Dimers self-associate through the LKR region to form homotetramers. **(C)** Proposed RNA-binding octamers mode. The yellow line represents the viral RNA strand wrapped around the helical supercomplex nucleocapsid protein. **(D)** SR-motif phosphorylation, i.e., nucleocapsid protein dissociation from RNP, exposes genomic RNA for replication.

Thus, the process of SARS-CoV-2 N protein-mediated RNP packaging may include several steps (Figure 4):

1. The CTD domain-mediated first dimerizes the N protein, and then homotetramer formation is mediated by the disordered C-terminal region (Ye et al., 2020);
2. The NTD domain mediates the binding of full-length N protein to genomic RNA through electrostatic interactions (Chang et al., 2014);
3. The N protein itself assembles to form an octamer in the shape of the letter “X,” and multiple octamers aggregate to form a complex with genomic RNA (Chen et al., 2007);
4. The M protein covers the outside of N + RNA condensate, and the C-terminal region of N protein interacts with M protein to promote the self-assembly of spiral RNP through electrostatic attraction interaction (Luo et al., 2006);
5. The SR region seems to be involved in regulating the whole process through phosphorylation;

## 3 N Protein and Immunity

### 3.1 N Protein Is a Key Immune Evasion Factor of SARS-Coronaviruses-2

Interferon (IFN), originally identified as a substance that “interferes” with *in vitro* viral replication, is a cytokine produced by immune cells. The first IFNs to be identified were classified as type I IFNs (IFN α/β and related molecules). There are two other types: type II IFN (IFN-γ) and type III IFNs (IFN-λ). Each IFN binds to one of three type-specific receptors (Zhang et al., 2008). Type I IFNs are the first line of defense against viruses and play a critical role in initiating the host antiviral response (Siu et al., 2014). The N protein can act as an antagonist of type I IFNs signals to hijack the host cell’s inherent immune system and help the virus escape the host immune response. Clinical data has shown that the type I IFNs response is disturbed during SARS-CoV-2 infection.

**FIGURE 5:**
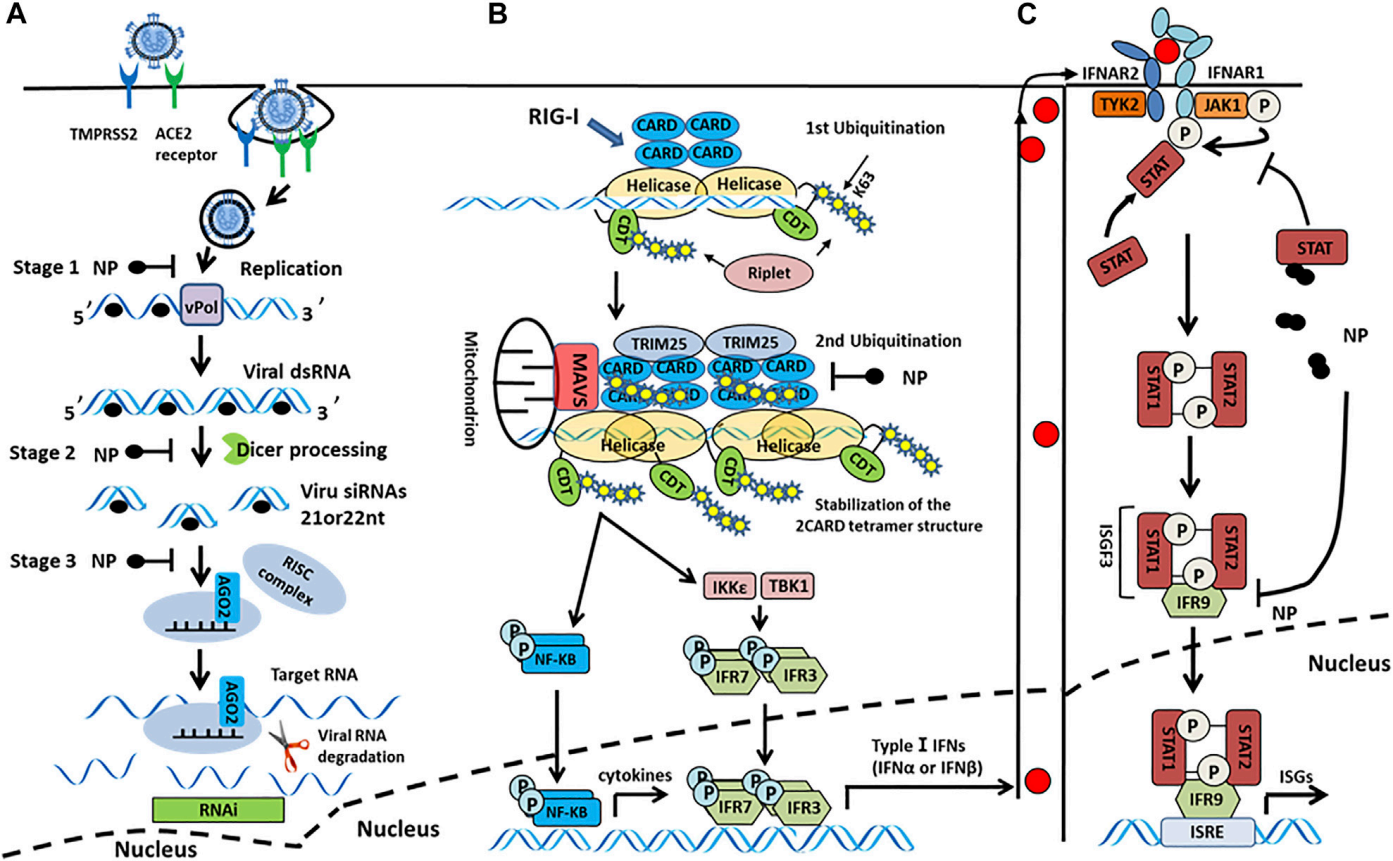
**(A)** Model of N protein suppression of RNAi in mammalian cells. In the initiation step, the single-stranded genomic RNA (gRNA) is protected by the N protein-after the entry and uncoating of coronaviral virions. The virus-derived dsRNA are generated during viral transcription and replication. In subsequent steps, these viral dsRNAs in infected cells could be protected by N proteins, which prevent the recognition and cleavage of viral dsRNA by Dicer and Ago2. In the last step, the N protein suppressed siRNA-induced RNA degradation. **(B)** N protein antagonizes type I IFN induction by interfering with TRIM25-mediated RIG-I ubiquitination. The CTD domain of RIG-I protein recognizes 5′ triphosphates at the end of dsRNA, and Riplet mediates two ubiquitination, one at K63-linked polyubiquitination at the end of dsRNA and the other ubiquitinate at CTDs that assemble along dsRNA. Subsequently, cooperative oligomerization of TRIM25 with the 2CARDs induces the second ubiquitination. The CTD of N protein interacts with TRIM25 to block RIG-I ubiquitination and activation. **(C)** N protein interacts with signal transducers and activators of transcription 1 and 2 (STAT1 and STAT2) and inhibits STAT phosphorylation, thereby reducing the subsequent nuclear translocation of the IFN-stimulated gene factor 3 (ISGF3) transcription complex and inhibiting the expression of IFN stimulated genes (ISGs), ultimately antagonizing type I IFN signaling.

SARS-CoV-2 N protein binds to RIG-I *in vitro*, inhibiting the type I IFN-β response induced by the overexpression of RIG-I pathway signaling molecules (e.g., MAVS, TBK1, and IKKε). This suggests that the N protein may inhibit type I IFN-β production by interfering with the initial targeting step-pattern recognition receptor that identifies RNA (PRR-RNA recognition) (Chen et al., 2020). The high cytosine phosphate guanosine (CpG) content of N protein may enable SARS-CoV-2 to enter the host cell and trigger the PRRs under different conditions (Di Gioacchino et al., 2021). Moreover, N protein interacts with STAT1 and STAT2, inhibits STAT phosphorylation, and inhibits IFN-induced nuclear translocation, thereby interfering with the antiviral response (Figure 5C) (Mu et al., 2020). Similarly, other HCoVs N proteins can also interfere with the production of type I IFNs. Studies have shown that SARS-CoV, MERS-CoV, and HCoV-OC43 N proteins promote viral replication by inhibiting TRIM25 to block RIG-I ubiquitination and activation and inhibiting IRF3 phosphorylation mediated type I IFN-β production (Kopecky-Bromberg et al., 2007; Hu et al., 2017; Beidas and Chehadeh, 2018; Chang et al., 2020). It is generally thought that SARS-CoV-2 N protein might interfere with the production of type I IFNs by inhibiting multiple signal analyses of RIG-I-like receptor pathways (Figure 5B).

It should be noted that type I IFNs can upregulate the gene expression and cellular levels at the receptor angiotensin-converting enzyme 2 (ACE2) of the cell invaded by SARS-CoV-2, and the increase of ACE2 receptor will lead to the aggravation of COVID-19 (Zamorano Cuervo and Grandvaux, 2020; Ziegler et al., 2020). Subsequent studies showed that hosts treated with type I IFNs exhibit effective antiviral capabilities, which can counteract the induction of ACE2 to restrain SARS-CoV-2 replication (Busnadiego et al., 2020). Therefore, despite the upregulation of viral replication caused by the upregulation of ACE2 expression, IFNs have an overall stronger antiviral effect.

In addition to antagonizing the type I IFN pathway, the SARS-CoV-2 N protein can also inhibit the cellular RNA interference (RNAi) process. RNAi is a process of gene silencing after eukaryotic transcription that enables host cells to have an antiviral immune function. To cope with this and escape the cellular immune response, many viruses promote their self-replication by encoding viral suppressors of RNAi (VSRs). Activity VSRs were first discovered in SARS-CoV N protein (Cui et al., 2015). Furthermore, the same highly conserved SARS-CoV-2 N protein can also act as a VSR in the host during the initial step (i.e., siRNA biogenesis) and the effect step (i.e., RNA-induced silencing complex assembly and target RNA cleavage) of RNAi process (Figure 5A). In summary, the SARS-CoV-2 N protein helps the virus escape the host’s immune response by interfering with the production of type I IFNs and acting as a VSR. Thus, for patients in the early stage of COVID-19, type I IFNs therapy has a certain effect.

### 3.2 N Protein Participates in the Host’s Excessive Inflammation Process

SARS-CoV-2 infection can trigger a “cytokine storm” to release a large amount of pro-inflammatory cytokines. Uncontrolled aggressive inflammation may cause multiple organs damage, which is one of the important reasons for the death of COVID-19 patients. The process mechanism is closely related to the lymphokine interleukin-6 (IL-6) and glycogen synthase kinase-3 (GSK-3).

#### 3.2.1 N Protein Activated IL-6 Upregulation Is Associated With Disease Severity

As a pleiotropic cytokine, IL-6 plays an important role in immune response, inflammation, and other multiple processes (Papanicolaou et al., 1998). In the COVID-19 patients’ cells, IL-6 and other peripheral blood inflammatory factors are elevated, and the expression level of IL-6 is positively correlated with the severity of COVID-19 (Liu et al., 2020). Meanwhile, high expression of IL-6 was also found in SARS patients (Hsueh et al., 2004). *In vitro* experiments have shown that the SARS-CoV N protein can directly interact with NF-κB, transport it to the nucleus, and ultimately upregulate IL-6 gene expression (Zhang et al., 2007). Because HCoV-OC43 N protein has also been reported to activate NF-κB (Lai et al., 2014), SARS-CoV-2 N protein may also further upregulate the expression of IL-6 by recognizing and activating the NF-κB factor, ultimately leading to inflammation.

#### 3.2.2 GSK-3 Mediated Phosphorylation of N Protein Enhances Virus Replication

Glycogen synthase kinase 3 (GSK-3: GSK-3α and GSK-3β) is a versatile serine/threonine kinase that mediates serine and threonine residues phosphorylation of downstream target molecules (Rana et al., 2021); GSK-3 includes two main functional domains: one is a substrate-binding domain, and the other is responsible for the phosphorylation of substrates. Phosphorylation of N protein is a necessary process for the transcription of the viral genome and that the phosphorylated N protein has an increased affinity for viral RNA (Liu et al., 2021), which facilitates RNA helicase DDX1 to turn the discontinuous transcription of CoVs into a continuous process (Wu et al., 2014). GSK-3 phosphorylates the similar substrate sequence of the N protein LKR domain between SARS-CoV and SARS-CoV-2, indicating that GSK-3 may be involved in the N protein phosphorylation. Indeed, after knocking out *GSK-3*, the phosphorylation of N protein was completely prevented (Chen et al., 2005). LiCl is a GSK-3 inhibitor. When the IC50 concentration of LiCl reaches 10 mM in 293T cells, it can inhibit N protein phosphorylation. Simultaneously, VeroE6 cells infected with SARS-CoV after treatment with a GSK-3 inhibitor showed that virus titer and cytopathic effects are reduced by 85% (Liu et al., 2021). Thus, GSK-3 is an important kinase in the phosphorylation of N protein and plays a key role in the virus’s life cycle. Therefore, targeting GSK-3 may be an option for COVID-19 treatment.

We carefully surveyed the mechanism of N protein in viral immune evasion. Antagonizing IFN production is an evasion innate immunity mechanism exploited by viruses in order to survive and replicate in host cells. The virus N protein interacts with TRIM25 through its C-terminal domain to suppress IFN promoter activity, but different types of viruses interact with different domains of TRIM25. For example, Influenza A virus NS1 protein interacting with the coiled-coil domain of TRIM25 results in the destruction of TRIM25 multimerization, while the V proteins of paramyxoviruses interact with the SPRY domain of TRIM25 (Gack et al., 2009; Sánchez-Aparicio et al., 2018). Structural analysis will be of value to clarify which domain of the SARS-CoV-2 N protein interacts with TRIM25. In addition, several potential ubiquitination sites in the MERS-CoV N protein have been revealed by the web-based server UbiSite (Chang et al., 2020), research on whether the SARS-CoV-2 N protein might also be ubiquitinated by TRIM25, and what possible mechanisms this ubiquitination might bring about, is very interesting. Current studies suggests that the phosphorylation of N protein may facilitate the disassembly of RNP in early stage of virus infection, which promotes the release of the viral genome in hosts for subsequent virus genome replication. Later, the phosphorylation of N protein is required to complete RNA packing and virus assembly. Therefore, the N protein-specific phosphorylation inhibitors may hamper virus replication in the early stage of viral infection (Law et al., 2003). GSK-3, a main N protein-phosphorylated kinase, has been targeted to screen a variety of inhibitors (Zhou et al., 2020; Snitow et al., 2021). However, GSK-3 is the key role for multiple cellular signaling pathways (Alfhili et al., 2020). The hindrance of non-specific GSK-3 inhibitors against normal cellular signaling is worth considering. N protein-specific phosphorylation inhibitors may be the solution to this problem. NF-κB is currently considered the main central regulator of SARS-CoV-2-mediated inflammatory responses (Kanan et al., 2021). It is possible that certain NF-κB binding sites exist on the N protein, which the activation of NF-κB. Blocking the interaction of the N protein and NF-κB may be a feasible drug treatment strategy. However, a handful of research focused on the identification of the binding sites within N protein or NF-κB. Most drug studies have focused on targeting NF-κB alone, which may lead to the specificity deficiency (Peddapalli et al., 2021). Therefore, more interactive information of N protein and NF-κB needs to be determined.

## 4 N Protein is a Target of Vaccine and Drug Development

### 4.1 N Protein Is a Potential Vaccine Candidate for SARS-Coronaviruses-2

At present, the vaccines against SARS-CoV-2 that have successfully entered phase III clinical or approved, mainly include mRNA vaccines, non-replicating viral vectors, protein subunit vaccines, and inactivated vaccines. Most are Spike-only vaccines. With the surge of global variants of concern (VOC), the risk of virus evasion has shapely risen (Aleebrahim-Dehkordi et al., 2022). Notably, many studies have reported the rapid decrease in neutralizing antibody levels among the vaccinated or virus-infected population (Ibarrondo et al., 2020; Long et al., 2020; Vabret, 2020; Koerber et al., 2022), causing the continuing decline in antibody-dependent vaccine protective efficacy. Therefore, stimulating potent and functional T-cell immunity are the current focus of vaccine design.

#### 4.1.1 N Protein-Elicited T-Cell Immune Response Is Associated With Severity of Disease

The N-specific T-cell response and epitopes have been sequentially demonstrated in cases infected with SARS-CoV-2 recently (Altmann and Boyton, 2020). The N-mediated T-cell immune response was considered to be associated with COVID-19 severity in the early infection stage. In some mild patients, the N-specific CD8^+^ T-cell response frequencies were significantly higher than the response frequencies of CD4^+^ T cells. Moreover, the proportion of N-specific CD8^+^ T cells was larger than that of S-specific. In contrast, no visible discrepancy was observed in severity (Peng et al., 2020b; Kared et al., 2021). Partial N-specific dominant epitopes display comparable immune response intensity compared to S protein (Peng et al., 2020b; Lee et al., 2021). An NP_105-113_ peptide was identified as the severity-related CD8^+^ T-cell immunodominant epitope (Peng et al., 2022). The corresponding NP_105_-specific CD8^+^ T cells (B7/N_105_) show high T cell receptor (TCR) diversity and expansion frequency in COVID-19 individuals’ blood samples. This may reshape the virus-susceptibility and severity in early infection (Lineburg et al., 2021). In addition, the shared epitopes of CD8^+^ T-cell responses were also mainly concentrated on N protein or ORF1ab, suggesting that N protein-mediated CD8^+^ T-cell responses possess a higher breadth among common individuals (Ferretti et al., 2020). In the convalescent stage, nearly 50% CD8^+^ T cells and 40% CD4^+^ T cells were triggered by non-S proteins including N protein. At this phase, different antigen-mediated CD4^+^ T cells present functional discrepancy. S-specific CD4^+^ T cells tend to be a circulating T follicular helper phenotype, and N-specific CD4^+^ T cells display T helper 1(Th1) memory phenotype bias (Sekine et al., 2020). The reactivation of Th1 memory CD4^+^ T cells is the key to CD8^+^ cytotoxic T-cell survival (Matloubian et al., 1994; Huang et al., 2007). The later inhibit the replication of viruses by recognizing and eliminating pathogen-infected cells. The functional loss or exhaustion of cytotoxic T lymphocyte is an important indicator of disease severity (Chen and John Wherry, 2020).

#### 4.1.2 N protein has Long-Lasting Immune Response

The long-term characteristics of T-cell immune responses are also the main concerns in vaccine design (Dan et al., 2021). Serum studies on SARS-CoV convalescent individuals have demonstrated that the N-elicited memory CD4^+^ T cells persisted in the donors’ blood for 6–11 years (Tang et al., 2011; Ng et al., 2016). The corresponding T-cell responses remain strong against the N protein of SARS-CoV after 17 years, and the SARS-CoV-2-specific cross-reactive responses were also observed (Le Bert et al., 2020), showing the long-lasting effects of N-elicited T-cell immunity. Similar SARS-CoV-2-specific T-cell cross-reactivity has been detected in 20–80% of unexposed healthy individuals (Braun et al., 2020; Nelde et al., 2021). Intriguingly, the N-specific cross-reactive frequencies in COVID-19 PCR-negative contacts were proportionately higher than that in PCR-positive contacts, revealing the intervention capacity of N-specific T-cell cross-reactivity on disease course (Kundu et al., 2022). The characterization of SARS-CoV-2 immunodominant epitopes further demonstrated that the conserved N-protein dominant epitopes are the main sites to initiate cross-reactivity (Lineburg et al., 2021).

Based on above preliminary analysis of N-specific T-cell pathogenic immune responses, developing a multi-antigen SARS-CoV-2 next-generation vaccine is a feasible solution to tackle the current pandemic. The N protein containing multi-antigen vaccine can not only reduce the risk of immune evasion caused by virus mutation but also increase the diversity of SARS-CoV-2-specific T-cell epitope recognition and elicit polyfunctional T-cell response in a wide range of patients. As the N protein is highly immunogenic and highly expressed in SARS-CoV-2, the immune response to N protein may be earlier than that to S protein (Burbelo et al., 2020), and the early-onset T-cell response of N protein containing vaccine may prevent the severity in the prime infection stage. However, several problems should not be overlooked: 1) in a study of severe patients, the SARS-CoV-2-specific T cells display strong expansion but low avidity and TCR clonality, including pre-existing cross-reactive memory CD4^+^ T cells. Such strongly expanded but low-avidity T-cell responses and low TCR clonality were also thought to be the reason for COVID-19 severity (Bacher et al., 2020). In view of this, it is necessary to evaluate the functional avidity degree among the vaccine-elicited T cells. 2) In SARS-CoV, Th2 bias CD4^+^ T-cell immune responses have been demonstrated in a mice trial, and host immune responses to SARS-CoV N protein resulted in severe pneumonia (Yasui et al., 2008). Although the current SARS-CoV-2–targeted CD4^+^ T-cell response is Th1 bias (Aleebrahim-Dehkordi et al., 2022), it still requires more clinical data and rigorous assessment for the N-protein-targeted candidate vaccine.

### 4.2 N Protein has the Potential to Develop a Cross-Prevention Coronaviruses Vaccine

The emergence of SARS-CoV in 2003 caused great economic loss and human deaths, and since late 2019, the homologous virus SARS-CoV-2 has been causing a global pandemic. Similar zoonotic variant CoVs may appear again in the future. Therefore, developing a vaccine that can cross-prevent mutated viruses is crucial and a long-term goal to defeat the viruses. The N protein is highly conservative and immunogenic, making it a suitable candidate for designing a vaccine. It also implies that it has the potential to develop a cross-prevention CoV vaccine in the future (Arthur et al., 1998; Kim et al., 2004; Zhao et al., 2005). Sergio C. Oliveira et al. predicted the main B and T cell epitopes of the SARS-CoV-2 N protein by bioinformatics. They found that residues 101–120 of the N gene sequence traverse the T cell cross-reaction regions of six HCoVs (except MERS-CoV) (Oliveira et al., 2020), suggesting that cross-prevention CoV vaccines are possible.

Furthermore, the sequence of T cells induced by N protein has species similarity. Murine splenocytes were analyzed for recognition of N-based peptide pools by enzyme-linked immunospot (ELISpot) assay. This revealed a T-cell epitope single region (residues 210–239: MANGGDAALALLLLDRLNQLESKMSGKGQ) to which T cells produced both interleukin-2 (IL-2) and gamma interferon (IFN-γ). Importantly, the sequence of this region is 100% identical to that of pangolin CoV and has an 86% homology with that of bat CoV (Dutta et al., 2020). Thus, the N protein is likely to induce immune cells that recognize other CoVs in future outbreaks. In summary, the N protein can design a cross-defense CoV vaccine, but the journey is still far away.

## 5 N Protein Is an Attractive Drug Target

The N protein is central to several critical events in virus production. Its highly druggable nature and rich antigenic determinants make it an excellent antiviral biomolecule, and it is considered a potential candidate for the design of new drugs (Ahmad et al., 2021). We summarized the three current therapeutic strategies for COVID-19, which target the HCoV N protein: 1) inhibiting the RNA binding of N-NTD; 2) interfering with the normal oligomerization of the N protein; and 3) decreasing the liquid–liquid phase separation of the N-protein.

### 5.1 Inhibiting the RNA Binding of N Protein

**FIGURE 6:**
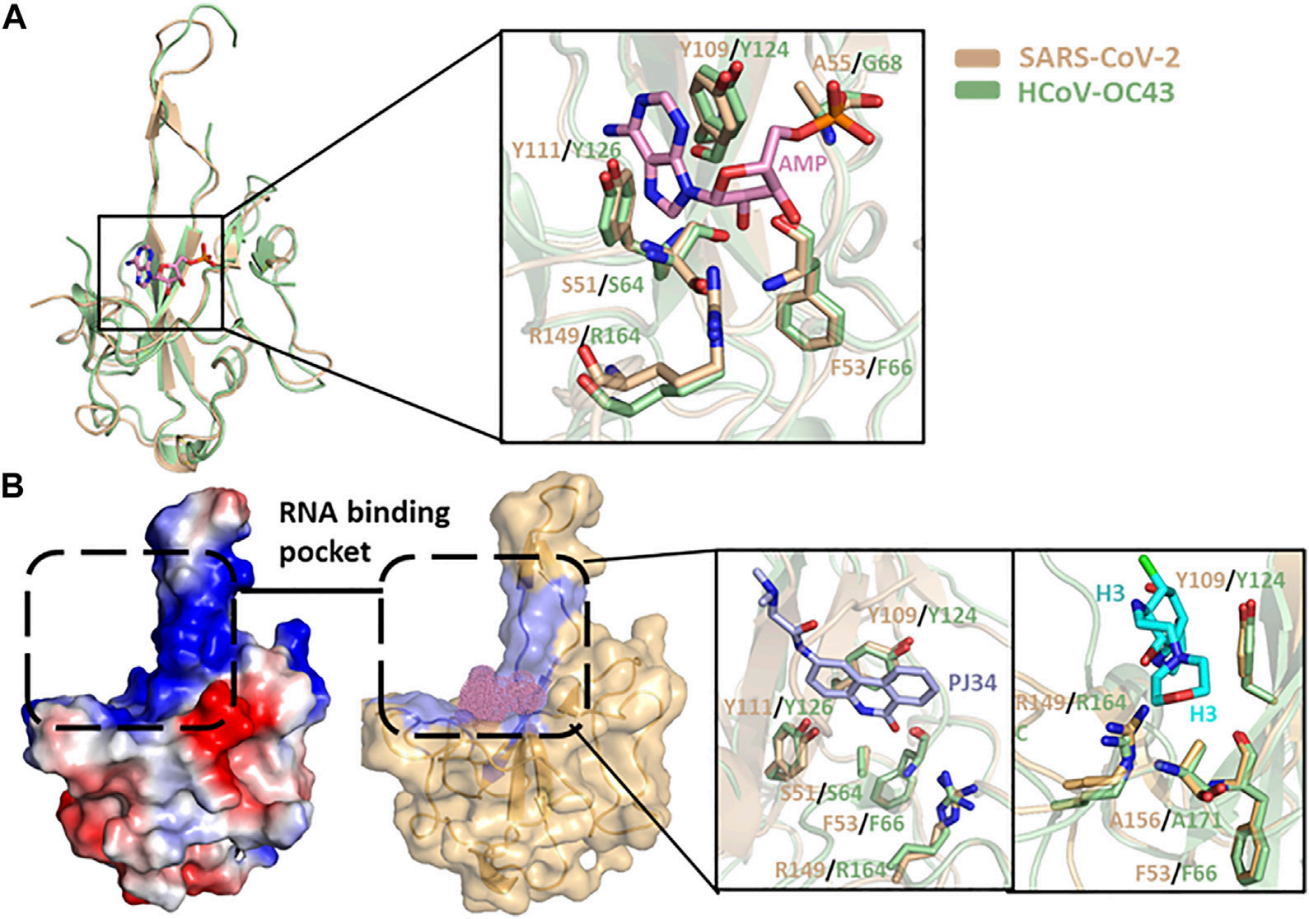
**(A)** N protein NTD structure superimposition of SARS-CoV-2 (wheat, PDB:7CDZ) and HCoV-OC43 (pale green, PDB: 4LI4). The protein-ligand interactive residues are shown in the right panel. **(B)** Electrostatic surface view and putative RNA-binding pocket of N protein NTD of SARS-CoV-2. The interactive positions of putative compound are shown as pink dots. The detailed interaction between PJ43/H3 and protein in RNA-binding pocket of NTD are shown in the right panel.

Different HCoV N protein NTDs have high folding similarities (Figure 3C) and are considered to act as RNA-binding domains. Lin et al. solved the complex structure of HCoV-OC43 and multiple ribonucleotide monophosphate, and identified the RNA-binding pockets of NTD domains and the key interactive residues involved in the binding of RNA mononucleotides (Lin et al., 2014). By superimposing the SARS-CoV-2 N protein’s NTD apo structure and HCOV-43 N protein’s NTD-AMP complex structure, it is possible to determine that the SARS-CoV-2 NTD resembles RNA-binding pocket mode and conserved RNA-binding residue arrangement with HCoV-OC43 (Figure 6A). Based on the HCoV-OC43 structure solved above, Lin et al. and Chang et al. virtually screened and identified two novel coronavirus N protein inhibitors: PJ34 (N-(6-oxo-5,6-dihydrophenanthridin-2-yl) (N,N-dimethylamino)acetamide hydrochloride) and H3 (6-Chloro-7-(2-morpholin-4-yl-ethylamino)quinoxaline-5,8-dione). Both compounds effectively reduced the N protein’s RNA-binding affinity and hampered viral replication (Lin et al., 2014; Chang et al., 2016). In the crystal structure of the complex formed by the two compounds with the HCoV-OC43 N protein NTD, both compounds occupy the RNA-binding pocket of the NTD. PJ34 interacts with Ser64, Phe66,Tyr124,Tyr126, and Arg164 via hydrogen bonding and π–π stacking, and corresponding residues interacting with H3 include Phe66,Tyr124,Arg164, and Ala171. Meanwhile, the homologous residues in SARS-CoV-2 NTD which may interact with H3 or PJ34 structure are located in the same position against HCoV-OC43, implying that the two compounds may still act on the same sites within SARS-CoV-2 N protein (Figure 6B and Table 3). In addition, PJ34 and H3 have their own drug benefits. H3 had a higher inhibitory effect on the RNA-binding activity of the HCoV-OC43 nucleocapsid. The viability of cell line was not affected when treated with PJ34 up to 20 μM alone for 24 h, indicating that PJ34 has a certain degree of safety to become a drug candidate for the treatment of COVID-19.

**TABLE 3:** Summary of compounds acting on HCoV N proteins.

Reference	Compound	Organism	Target Site	Key Residues Interaction	Homologous Key Residues of SARS-CoV-2 Nucleocapsid	Target Determination Method
Lin et al. (2014)	PJ34	HCoV-OC43	RNA binding pocket	Ser64, Phe66, Tyr124,Tyr126, Arg149	Ser51, Phe53, Tyr109, Tyr111, Arg164	Co-crystallization
Chang et al. (2016)	H3	HCoV-OC43	RNA binding pocket	Phe66, Tyr124, Arg164, Ala171	Phe53, Tyr109, Arg149, Ala156	Co-crystallization
Muhseen et al. (2021)	glycyrrhizin	SARS-CoV-2	RNA binding pocket	Arg88, Thr91, Arg93, Tyr109,Arg149	—	Molecular Docking
Ren et al. (2022)	lycorine	SARS-CoV-2	RNA binding pocket	Arg88, Thr91, Arg93, Arg107, Tyr109, Tyr111, Arg149	—	Molecular Docking
	emetine					
	cephaeline					
Bhowmik et al. (2020)	simeprevir	SARS-CoV-2	RNA binding pocket	Thr91, Arg93	—	Molecular Docking
	grazoprevir			Tyr109, Tyr111		
Mishra et al. (2021); Nishimura et al. (2021)	catechin gallate allocatechin gallate		ND	ND	—	Molecular Docking
Bhowmik et al. (2020)	P3	MERS-CoV	NTD-Dimeric-site	Monomer 1: Vla41, Gly104, Thr105, Thr148 Monomer 2: Trp43, Asn66, Ser69, Thr70, Asn77, F135	Monomer 1: Ala50, Gly114, Thr115, Thr137 Monomer 2: Trp52, Asn68, Asn75, Ser78	Co-crystallization
Ahamad et al. (2020)	4E1RCAT	SARS-CoV-2	CTD-Dimeric-site	Arg277, Lys261 and Tyr333	—	Molecular Docking
	Silmitasertib			Arg277, Asn285 and Phe286		Molecular Docking
	TMCB			Arg277		Molecular Docking
	Sapanisertib			Lue331 and Tyr333		Molecular Docking
	Rapamycin			Arg277, Glu283, Asn285, Tyr333		Molecular Docking
Iserman et al. (2020); Seim et al. (2021)	Lipoic acid	SARS-CoV-2	ND	ND	—	Phase separation assays
	1,6-hexanediol					

*ND, not determined.

Computer-aided prediction of drug-binding sites and screening is also a powerful tool for N protein-directed drug identification and repurposing. Several groups have screened novel candidate drugs targeted for RNA-binding sites of N-NTD via silico studies. Bhowmik et al. reported that SimePrevir and GrazopRevir, which were identified from natural antiviral compounds and synthetic antiviral drugs, displayed good docking results for N proteins through virtual screening (Bhowmik et al., 2020). Ren et al. obtained three alkaloid compounds (lycorine, emetine, and cephaeline) from DrugBank 5.0 and the anti-infection database in molecular docking, and evaluated their binding affinity to the N protein by surface plasmon resonance (SPR) (Ren et al., 2022). Their results showed that, of the three alkaloid compounds, emetine had the strongest binding activity to the N protein. Muhseen et al. identified a multi-target drug, glycyrrhizin, from the MPD3 phytochemical database: glycyrrhizin. In docking results, glycyrrhizin not only had a very stable binding mode with the RNA-binding pocket of the NTD domain but also acted on main protease and papainlike protease to form strong affinity complexes (Muhseen et al., 2021). Moreover, the predicted residues in the RNA-binding pocket interacting with glycyrrhizin were significantly more than those that interacted with predicted alkaloid compounds, or SimePrevir and GrazopRevir (Table 3).

### 5.2 Interfering With the Oligomerization of N Protein

**FIGURE 7:**
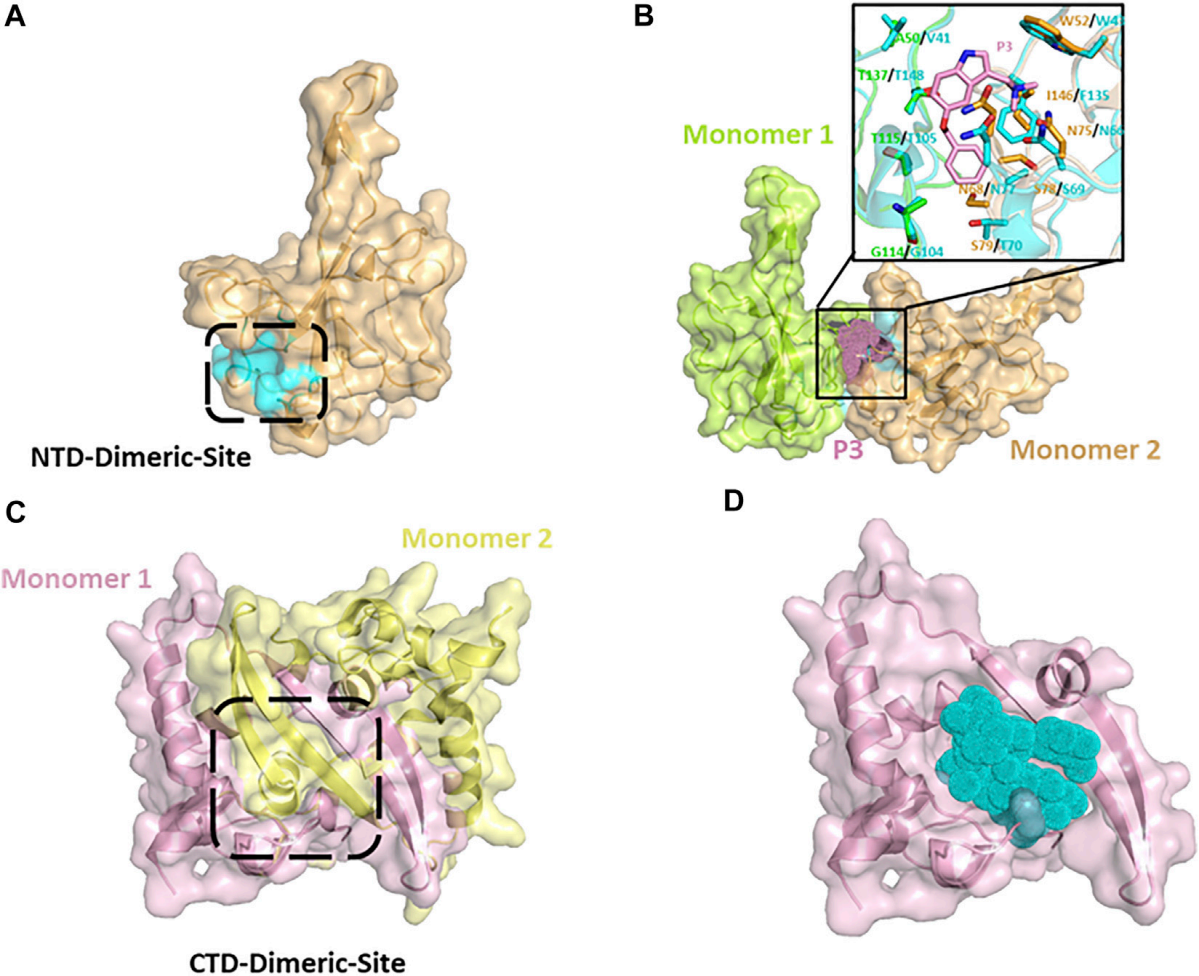
**(A)** Putative NTD-Dimeric-Site (cyan surface) of SARS-CoV-2 N protein derived from MERS-CoV-P3 complex-structure (PDB: 6KL6). **(B)** Putative abnormal dimer of SARS-CoV-2 N protein NTD predicted by superimposing of MERS-CoV-P3 complex (cyans, PDB:6KL6) with SARS-CoV-2 apo structure (Monomer 1: chartreuse, Monomer 2: wheat). The interactive positions of putative dimerization inducing compounds (pink dots) are located between Monomer 1 and Monomer 2. **(C)** CTD dimer of SARS-CoV-2 N protein (6WJI). **(D)** Putative SARS-CoV-2-compounds complex.

Small molecule-mediated protein-protein interaction stabilization (PPIS) is a potential strategy to regulate physiological functions or treat cancer and virus infection via changing the oligomerization equilibrium of target proteins. Several studies have successfully identified antiviral compounds to prevent RNP formation of nucleocapsid using PPIS strategy (Gerritz et al., 2011; White et al., 2018). Shan-Meng Lin et al. identified an NTD-dimer stabilizer P3 (5-benzyloxygramine) of MERS-CoV N protein from Acros and ZINC drug databases in the screening of anti-MERS-CoV drugs. P3 could induce and stabilize abnormal dimerization of N protein *in vitro* or cellular levels. In the crystal structure of the resolved MERS-CoV N protein NTD-P3 complex, P3 binds directly to the non-native dimer interface (CTD-Dimeric-Site) of N protein NTD (Figure 7A), and simultaneously interacts with dimeric monomer 1 residues (Val41, Gly104, Thr105, Thr148) and monomer 2 residues (Trp43, ASN66, ASN68, Ser69, Thr70, Phe135) to mediate the dimerization (Figure 7B and Table 3). In superimposing of the SARS-CoV-2 NTD structure with MERS-CoV NTD-P3 complex, except for a few residues (I146/F135, S79/T70), most of the homologous P3-interacting residues of SARS-CoV-2 N protein are located in the same spatial arrangement (Figure 7B), indicating that P3 may be a promising candidate drug for COVID-19 with the resembled target site and antivirus mechanism. Similar potential drugs have been found in computer virtual screening based on N protein abnormal oligomerization of SARS-CoV-2. Yadav et al. screened three ligands, including Zidovudine and two Asinex ligands (5817,6799), from Asinex and PubChem databases through high-throughput screening and extra precision docking. Both ligands were simultaneously attached to two N protein molecules in docking processing and formed residue interactions with each molecule. Moreover, Zidovudine showed more stable interactions in molecular simulations than the other two Asinex ligands (Yadav et al., 2021). An early antiviral study on Catechins against SARS-CoV reported that catechin gallate (CGs) and gallocatechin gallate (CCGs) significantly inhibit the binding of SARS-CoV N protein to RNA (Roh, 2012). Catechins are phenolic active substances extracted from natural plants such as tea. Recent study has confirmed that catechin mixture reagent still possessed the inactivation capacity against SARS-CoV-2 in a dose-dependent manner (Nishimura et al., 2021). The computer prediction of catechin target sites shows that catechin may be a multi-target drug and not act directly on the RNA-binding pocket of NTD to block RNA-binding. Therefore, the detailed anti-viral mechanism of catechins needs to be further confirmed (Mishra et al., 2021).

As we mentioned in Section 2.2, the N protein C-terminal is responsible for oligomerization in the formation of RNP. The CTD (254–364) of SARS-CoV-2 N protein is the basic dimeric unit of RNP and C-tail (364–419), which may be involved in the higher oligomeric states. Thus, it is a feasible therapeutic strategy to hamper the normal oligomerization of the N protein by developing competitive compounds or peptides. It was found that a β-sheet in the C-tail of HCoV-229E N protein may mediate the formation of high polymerization N proteins. The pharmaceutical screening against the C-tail of influenza viruses and the HCoV-229E N protein confirmed that small-molecule compounds and competitive peptides could hinder the formation of N protein high polymerization and inhibit viral replication (Shen et al., 2011). Ye et al. also predicted a α-helix in residues 400–416 of the SARS-CoV-2 C-tail region, indicating the possibility of introducing competitive peptides in SARS-CoV-2 drug design (Lo et al., 2013; Ye et al., 2020). According to recent studies, the CTD of the SARS-CoV-2 N protein forms stable dimers in crystal packing and solution (Yang et al., 2021; Zinzula et al., 2021). A protrusion composed of two β-sheets and a short α helix from each monomer extends into the hydrophobic surface of the opposite monomer (Figure 7C). Recently, Ahamad et al. identified five possible inhibitors of CTD-targeted dimerization based on the solved CTD structure (Table 3) (Ahamad et al., 2020). In the docking model, each inhibitor could occupy the hydrophobic core of the CTD monomer to block the assembly of the CTD dimer (Figure 7D). The researchers used structure-based calculations to elucidate the antivirus mechanism of these drugs, which could help the pandemic of COVID-19 in the future.

### 5.3 Altering Liquid-Liquid Phase Separation of N Proteins

Proteins, nucleic acids, or mixtures of them could form in a liquid-liquid phase separation (LLPS), (similar to the process of mixing water and oil as we understand). Many highly contagious RNA virus nucleocapsid proteins coagulate with the viral genome under physiological conditions to form LLPS. However, LLPS of the SARS-CoV-2 N protein was a handful reported (Iserman et al., 2020). LLPS and phase transition are modulated by numerous factors (Perdikari et al., 2020; Dang et al., 2021). Zinc ions (Zn^2+^) promote the formation of droplets, suggesting that LLPS might be related to electrostatic interactions. At low pH (∼5.5) conditions, the RNA-protein mixture reaction’s turbidity increased and persisted over time, but at a pH = 7.4, the turbidity almost disappeared. HCoVs have a strict requirement on pH and only survive at a neutral pH 6.7–7.7. We speculate that the HCoVs N protein changes its dimerization structure after binding to RNA in lower pH conditions, causing it to be refolded into higher-order polymers, and during this process, RNA may be wrapped inside the molecular interior of the N protein to be protected. Between 33°C (upper respiratory tract temperature) and 40°C (fever), as the temperature increases, LLPS is more likely to be formed between RNA and N protein (Iserman et al., 2020). This may be a self-protection behavior performed by the virus through LLPS in response to high temperatures. At present, no reports on *in vitro* experiments, have shown that the affinity of SARS-CoV-2 N protein to RNA is related to the specificity of the genomic RNA primary sequence. However, the LLPS is selective for RNA sequences. The 5′- and 3′-ends of SARS-CoV-2 genomic RNA can promote LLPS, whereas the middle partial sequence can inhibit LLPS (Iserman et al., 2020; Seim et al., 2021). Meanwhile, LLPS is a promising target for drug screening to hinder virus replication. Iserman et al. tested three compounds’ LLPS regulation effects, and confirmed that they regulate the LLPS of viruses in different ways, including lipoic acid, which interferes with hydrophobicity; 1,6-Hexanediol, which regulates protein-protein interaction; and kanamycin, which regulates protein-RNA interaction (Iserman et al., 2020). The elucidation of the active mechanism of these compounds on LLPS provides strong support for the design and testing of subsequent new drugs.

## 6 Prospects

SARS-CoV-2 causes COVID-19, a disease that became a global pandemic and has seriously threatened global health. Scientists have been following three main strategies to fight the COVID-19 pandemic: vaccines, neutralizing antibodies, and antiviral drugs, but no specific therapy has been found.

A possible SARS-CoV-2 N protein self-assembly model to form RNPs was proposed by sorting out most current research on the molecular mechanism of the self-assembled RNP of HCoVs. However, this is only a speculation model, and there are still many questions to be clarified by high-level evidence. Many experimental studies are still needed to clarify the interaction between SARS-CoV-2 N protein and RNA.

We also summarized the N protein-mediated mechanism of virus evasion and the inflammatory response. However, the physiological activities of the virus in the host are complicated, the regulation of the signaling pathway of N protein is complex, and whether the N protein will cooperate with other functional proteins co-regulate the life cycle is not completely understood. For example, the nonstructural protein (nsp13) in SARS-CoV-2 also has a strong affinity for RNA and contains a typical helicase domain. N protein has also been reported to bind RNA helicase. Does this binding affect the unwinding process of nsp13? How does the virus interact with various substances in the human after invading the hosts? These questions remain to be fully elucidated.

Finally, we addressed the feasibility and advantages of the N protein as a potential vaccine and drug target for COVID-19. Amid natural infection of viruses, CD8^+^ T-cell immune response induced by N protein in the early stage of COVID-19 may be a potent factor in inhibiting the course development of disease. A reasonable explanation is that the highly concentrated epitopes of N protein can trigger a frequent T-cell immune response. Introducing N-protein antigens into vaccine design is a powerful means to prevent severity transformation in patients. Meanwhile, considering the conservation of the N protein sequence in evolution, the low mutation-related improvement of epitope recognition rate can enhance the durability of vaccine T-cell immune response. However, introducing N protein antigens into vaccines an understanding of the inflammatory response caused by excess N protein. Therefore, using non-full-length N-protein antigens is a resonable approach, which may preserve high response epitopes, thus reducing the excessive immune response. Recent pharmaceutical research studies primarily focused on S proteins or proteases (3C-like, papain-like). The resulting side effects and cellular cytotoxicity inducing by viral proteases targeted inhibitors’ non-specifical affection on human homologous protease is a concern worthy of researchers’ attention. The conserved N protein-targeted drugs not only have the advantages of addressing the above drug specificity problem, but also can reduce the virus’ drug resistance due to its conserved sequence. Early studies of HCoV drug candidates have identified some N-protein targeting agents including JP43, H3, and P3. Through a comparative analysis based on structure and sequence, we preliminarily confirmed the repurposing possibility of such drugs and a variety of computer-assisted screening N-protein targeting drugs. However, few cell pathway interaction inhibitors specific to N protein have been developed, which may be due to the lack of structural understanding of the interactive sites of pathway proteins, leading to the inability to accurately design site-specific drugs molecules. Therefore, the N protein-related signaling pathways summarized in this review can provide broad ideas for the design of N protein-specific new drugs.
